# Ten-year trends in lipid management among patients after myocardial infarction in South Korea

**DOI:** 10.1371/journal.pone.0304710

**Published:** 2024-10-03

**Authors:** Seok Oh, Kyung Hoon Cho, Min Chul Kim, Doo Sun Sim, Young Joon Hong, Ju Han Kim, Youngkeun Ahn, Sang Yeub Lee, Min-Ho Shin, Weon Kim, Myung Ho Jeong

**Affiliations:** 1 Department of Cardiology, Chonnam National University Hospital, Gwangju, Republic of Korea; 2 Department of Cardiology, Chonnam National University Medical School, Gwangju, Republic of Korea; 3 Chung-Ang University Gwangmyeong Hospital and Department of Internal Medicine, Division of Cardiology, Chung-Ang University College of Medicine, Gwangmyeong, Republic of Korea; 4 Department of Preventive Medicine, Chonnam National University Medical School, Hwasun, Republic of Korea; 5 Department of Internal Medicine, Division of Cardiology, Kyung Hee University Hospital, Kyung Hee University, Seoul, Republic of Korea; 6 Department of Cardiology, Gwangju Veterans Hospital, Gwangju, Republic of Korea; Baylor Scott and White, Texas A&M College of Medicine, UNITED STATES

## Abstract

**Background:**

Dyslipidemia is an important risk factor for acute myocardial infarction. However, real-world data on its prevalence and lipid management trends for Korean patients with acute myocardial infarction are limited. This study aimed to determine the 10-year temporal trends in dyslipidemia prevalence and lipid management in this patient population.

**Methods and findings:**

The study used a merged database of two nationwide observational cohorts (2011–2020) that included 26,751 participants. The primary endpoints were the achievement rates of the (1) absolute low-density lipoprotein cholesterol (LDL-C) target of <70 mg/dL (<1.8 mmol/L), (2) relative LDL-C target reduction of >50% from the baseline, (3) absolute or relative LDL-C target (American target), and (4) both absolute and relative LDL-C targets (European target).

The dyslipidemia prevalence increased from 11.1% to 17.1%, whereas the statin prescription rate increased from 92.9% to 97.0% from 2011 to 2020. The rate of high-intensity statin use increased from 12.80% in 2012 to 69.30% in 2020. The rate of ezetimibe use increased from 4.50% in 2016 to 22.50% in 2020. The high-intensity statin and ezetimibe prescription rates (0.20% to 9.30% from 2016 to 2020) increased gradually. The absolute and relative LDL-C target achievement rates increased from 41.4% and 20.8% in 2012 to 62.5% and 39.5% in 2019, respectively. The American (45.7% in 2012 to 68.6% in 2019) and European (16.5% in 2012 to 33.8% in 2019) target achievement rates also increased.

**Conclusions:**

The adoption of lipid management guidelines in clinical practice has improved. However, continued efforts are needed to reduce the risk of recurrent ischemic events.

## Introduction

Atherosclerotic cardiovascular disease (ASCVD) is the leading cause of mortality globally, with an estimated 17 million deaths annually [1,2]. Acute myocardial infarction (AMI) requires adequate medical treatment and timely revascularization. The incidence of AMI has been decreasing in European countries and the United States, and this may be associated with the implementation of innovative preventive medical protocols and a parallel improvement in risk factor management [3]. By contrast, the incidence has been steadily increasing in several countries around the world, including South Korea, leading to a considerable socioeconomic burden [4]. Patients with AMI have higher risks of adverse cardiovascular events. Therefore, secondary prevention is required after the index AMI to preclude adverse events, including mortality, left ventricular dysfunction, and heart failure [5].

Dyslipidemia has been established as one of the modifiable risk factors of ASCVDs [6,7], prompting the recommendation of the control of serum lipid profiles by several guidelines developed in Europe and the United States [8–10]. Low-density lipoprotein cholesterol (LDL-C) is the recommended primary target for lipid management [11]. Regular amendments to these guidelines based on the results of clinical research have culminated in a consensus to heighten LDL-C reduction efforts [12,13].

For AMI, lipid management with lipid-lowering agents (LLAs) is important to reduce the chances of adverse cardiovascular events and improve survival. Thus, periodic revisions of lipid management guidelines may shape treatment patterns and clinical outcomes. However, real-world data on the temporal trends in dyslipidemia prevalence and lipid management in Korean patients with AMI are limited. Therefore, we aimed to evaluate the trends in dyslipidemia and lipid management strategies between 2011 and 2020 among Korean patients with AMI.

## Methods

### Ethics statement

The present study was conducted according to the ethical standards of the World Medical Association’s Declaration of Helsinki. This study was approved by the Institutional Review Board of Chonnam National University Hospital (IRB No. CNUH-2022-038). The authors confirm that patient consent is not applicable to this article. This study is a retrospective analysis of the de-identified database (the KAMIR-NIH and KAMIR-V cohorts); therefore, the institutional review board did not require informed consent from the participants.

### Data sources and study design

All epidemiological and clinical data were obtained from the Korean Acute Myocardial Infarction Registry (KAMIR), and the authors collected de-identified data for research purposes between July 11, 2023, and September 1, 2023. The KAMIR, a nationwide multicenter observational cohort [14], provides real-world de-identified data on the demographics, treatment strategies, and treatment outcomes of Korean patients hospitalized overnight for AMI. The KAMIR-National Institutes of Health (KAMIR-NIH, also known as KAMIR-IV) registry was maintained from November 01, 2011, to December 31, 2015, whereas the KAMIR-V registry was followed from January 01, 2016, to June 30, 2020. The KAMIR-NIH and KAMIR-V included 20 and 43 percutaneous coronary intervention (PCI)-capable tertiary centers, respectively. Their protocols were approved by the institutional review boards of each participating institution [15].

**Fig 1:**
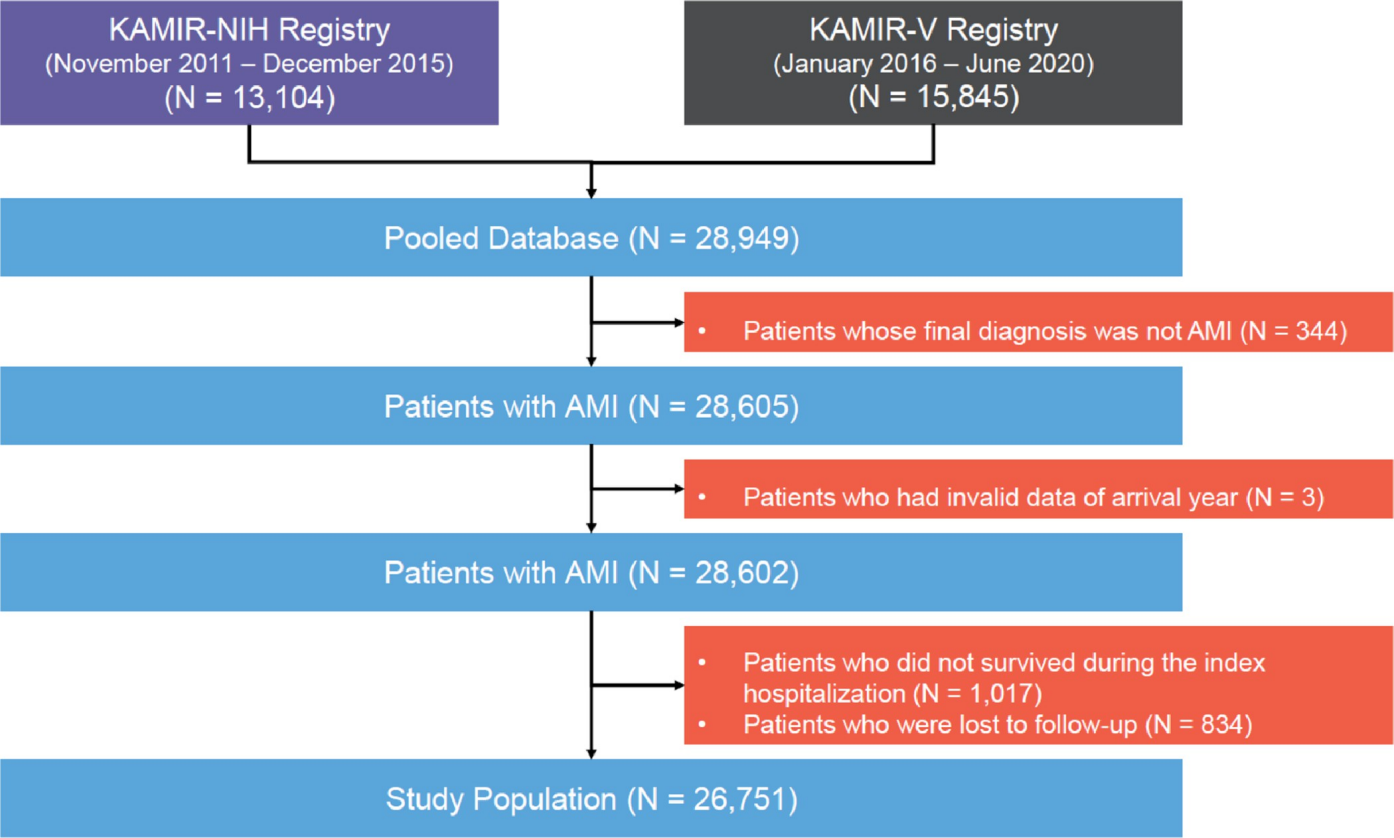
Study flowchart of the study population selection. AMI, acute myocardial infarction; KAMIR-NIH, Korea Acute Myocardial Infarction Registry-National Institutes of Health; KAMIR-V, Korea Acute Myocardial Infarction Registry-V.

Of the 28,949 patients whose data were retrieved from the KAMIR-NIH and KAMIR-V registries, we excluded (1) those without AMI as their final diagnosis, (2) those with invalid data on the arrival year, (3) those who did not survive during the index hospitalization, and (4) those lost to follow-up. Therefore, 26,751 participants were included in this study. To examine the baseline characteristics, all the participants were classified into two groups depending on their statin prescriptions. The patients were further divided into three subgroups according to the statin intensity. **Fig 1** shows a flowchart of the study scheme.

### Study definitions and clinical outcomes

Standardized definitions determined by the steering committee associations for both registries were used for all the covariate items. The prevalence of dyslipidemia was based on its history, irrespective of the presence of LLAs. The LLA-naïve patients had a history of dyslipidemia but did not have any exposure to LLAs before admission. These definitions were based on self-reported information or medical records.

The LLAs available for our statistical analysis included statins, ezetimibe, fibrate, and omega-3 fatty acids. Data regarding ezetimibe prescriptions could only be accessed from the KAMIR-V registry (2016–2020) database. In this study, high-intensity statins were atorvastatin ≥40 mg per day or rosuvastatin ≥20 mg per day, according to current guidelines.^8^

The absolute LDL-C target was defined as a concentration of <70 mg/dL (<1.8 mmol/L), whereas the relative LDL-C target was defined as a reduction of >50% from the baseline. These definitions were based on the 2018 American College of Cardiology/American Heart Association (ACC/AHA) and the 2016 European Society of Cardiology/European Atherosclerosis Society (ESC/EAS) cholesterol guidelines [8,16]. Despite more recent advances in lipid management [10,17], we took the time required for the collection of these data into account and used these two guidelines for our analysis.

LDL-C has been considered the primary treatment target for lipid management in patients with ASCVD [11]. Therefore, the primary endpoints were the achievement rates of the LDL-C goals, which are as follows: (1) achieving only the absolute LDL-C target goal, (2) achieving only the relative LDL-C target goal, (3) achieving either an absolute or relative LDL-C target goal, and (4) achieving both absolute and relative LDL-C target goals. We labeled endpoint 3 as the American goal and endpoint 4 as the European goal.

### Statistical methods

All statistical analyses were performed using both IBM SPSS (version 25.0; IBM Corp., Armonk, NY, USA) and SAS software version 9.1.3 (SAS Institute Inc., Cary, NC, USA). Continuous data are expressed as means and standard variations, whereas categorical data are expressed as frequencies and percentages. The continuous data were analyzed statistically using the two-tailed Student’s *t*-test or one-way analysis of variance. The categorical data were analyzed using Pearson’s chi-squared test, Fisher’s two-by-two exact test, or the Cochran–Mantel–Haenszel test. We determined the trends for each calendar year. Gradual trends in the dichotomous variables were analyzed using the Cochran–Armitage asymptotic test. *p*-values <0.05 indicated statistical significance.

Multivariable logistic regression analysis was used to examine the association among statin prescription, statin intensity, and the achievement of LDL-C target goals. Different models were used to assess the robustness and consistency of our findings. Model 1 indicated the crude hazard ratios (HRs) with 95% confidence intervals (CIs), whereas Models 2 and 3 indicated the partially adjusted HRs with 95% CIs. Model 2 was adjusted for age (≥75 versus <75 years) and sex (male sex versus female sex), whereas Model 3 was adjusted for all components in Model 2 along with the use of emergency medical services, Killip functional class (Killip classes III–IV versus I–II), body mass index (≥25 versus <25 kg/m^2^), smoking status, medical history, family history of coronary artery disease, and serum creatinine concentration (≥1.5 versus <1.5 mg/dL). Model 4, the completely adjusted model, was adjusted for all the components in Model 3 along with post-discharge medications, left ventricular ejection fraction (LVEF) (<40 versus ≥40%), and the final diagnosis (ST-elevation myocardial infarction [STEMI] versus non-STEMI). Model 4 was considered the main model for interpretation.

To suppress the pretreatment effect of dyslipidemia, we applied identical statistical analyses to the overall population and LLA-naïve participants.

## Results

### Baseline characteristics

**Table 1** and **S1 Text** summarize the demographic characteristics, prevalence of comorbidities, and healthcare use of the study cohort and the LLA-naïve participants, respectively. Among the 26,751 participants included in statistical analyses, the mean age was 63.6 years, 76.2% were men, 47.3% were diagnosed with STEMI, and 52.7% were diagnosed with non-STEMI.

**Table 1:** Baseline characteristics of the participants in the study cohort.

	Overall population	Prescription fills of statins			Patterns of statin prescriptions stratified by statin intensity		
	(N = 26,751)	Statin group	No-statin group	*p*-value between two groups	Moderate-intensity statins (N = 13,325)	High-intensity statins (N = 11,967)	*p*-value between three groups
		(N = 25,292)	(N = 1,459)				
**Age ≥75 years**	5,964 (22.3)	5,529 (21.9)	435 (29.8)	<0.001	3,348 (25.1)	2,181 (18.2)	<0.001
**Age, years**	63.63 ± 12.43	63.49 ± 12.40	66.11 ± 12.76	<0.001	64.64 ± 12.45	62.20 ± 12.21	<0.001
**Male sex**	20,379 (76.2)	19,358 (76.5)	1,021 (70.0)	<0.001	9,826 (73.7)	9,532 (79.7)	<0.001
**Smoking history**	15,042 (57.9)	14,365 (58.5)	677 (48.4)	<0.001	7,234 (55.8)	7,131 (61.4)	<0.001
**Use of EMS**	4,701 (17.6)	4,465 (17.7)	236 (16.2)	0.149	2,300 (17.3)	2,165 (18.1)	0.025
BMI, kg/m^2^	24.22 ± 3.35	24.24 ± 3.34	23.82 ± 3.50	<0.001	24.00 ± 3.34	24.51 ± 3.31	<0.001
BMI ≥25 kg/m^2^	9,361 (37.1)	8,925 (37.3)	436 (33.4)	0.005	4,316 (34.4)	4,609 (40.4)	<0.001
**Killip class III–IV**	2,632 (9.9)	2,364 (9.4)	268 (18.4)	<0.001	1,354 (10.2)	1,010 (8.5)	<0.001
**Past medical history**							
** Hypertension**	13,381 (50.0)	12,574 (49.7)	807 (55.3)	<0.001	6,940 (52.1)	5,634 (47.1)	<0.001
** Diabetes mellitus**	7,403 (27.7)	6,860 (27.1)	543 (37.2)	<0.001	3,827 (28.7)	3,033 (25.3)	<0.001
** Dyslipidemia**	3,518 (13.2)	3,365 (13.3)	153 (10.5)	0.002	1,692 (12.7)	1,673 (14.0)	<0.001
** Dyslipidemia on treatment**	2,679 (10.1)	2,559 (10.2)	120 (8.3)	0.019	1,327 (10.0)	1,232 (10.4)	0.040
** Prior CAD**	3,933 (14.7)	3,627 (14.3)	306 (21.0)	<0.001	2,223 (16.7)	1,404 (11.7)	<0.001
** Prior CVA**	1,708 (6.4)	1,582 (6.3)	126 (8.7)	<0.001	929 (7.0)	653 (5.5)	<0.001
**Family history of CAD**	1,959 (7.5)	1,886 (7.7)	73 (5.2)	0.001	832 (6.4)	1,054 (9.1)	<0.001
**Serum creatinine ≥1.5 mg/dL**	2,547 (9.5)	2,242 (8.9)	305 (20.9)	<0.001	1,356 (10.2)	886 (7.4)	<0.001
** Serum creatinine, mg/dL**	1.13 ± 1.69	1.11 ± 1.69	1.45 ± 1.74	<0.001	1.14 ± 1.35	1.08 ± 2.00	<0.001
**LVEF <40%**	3,028 (11.7)	2,769 (11.3)	259 (19.0)	<0.001	1,550 (12.0)	1,219 (10.4)	<0.001
** LVEF, %**	52.46 ± 10.98	52.58 ± 10.87	50.26 ± 12.53	<0.001	52.14 ± 11.07	53.06 ± 10.63	<0.001
**STEMI as a final diagnosis**	12,644 (47.3)	12,098 (47.8)	546 (37.4)	<0.001	6,157 (46.2)	5,941 (49.6)	<0.001
**PCI utilization**	24,541 (91.7)	23,518 (93.0)	1,023 (70.1)	<0.001	12,075 (90.6)	11,443 (95.6)	<0.001
**Post-discharge medications**							
** Aspirin**	26,612 (99.5)	25,206 (99.7)	1,406 (96.4)	<0.001	13,267 (99.6)	11,939 (99.8)	<0.001
** P2Y12 inhibitors**	26,537 (99.2)	25,147 (99.4)	1,390 (95.3)	<0.001	13,221 (99.2)	11,926 (99.7)	<0.001
** Beta-blockers**	21,331 (79.7)	20,418 (80.7)	913 (62.6)	<0.001	10,722 (80.5)	9,696 (81.0)	<0.001
** RAAS inhibitors**	20,711 (77.4)	19,840 (78.4)	871 (59.7)	<0.001	10,522 (79.0)	9,318 (77.9)	<0.001
** Fibrates**	164 (0.6)	140 (0.6)	24 (1.6)	<0.001	79 (0.6)	61 (0.5)	<0.001
** Omega-3 fatty acids**	554 (2.1)	528 (2.1)	26 (1.8)	0.425	298 (2.2)	230 (1.9)	0.321
**Laboratory results at the initial stage**							
** TC, mg/dL (mmol/L)**	177.97 ± 49.18 (4.60 ± 1.27)	178.78 ± 49.15 (4.62 ± 1.27)	163.27 ± 47.25 (4.22 ± 1.22)	<0.001	174.06 ± 50.91 (4.50 ± 1.32)	184.28 ± 46.43 (4.77 ± 1.20)	<0.001
** TG, mg/dL (mmol/L)**	140.51 ± 115.03 (3.63 ± 2.97)	141.10 ± 115.61 (3.65 ± 2.99)	129.26 ± 102.90 (3.34 ± 2.66)	<0.001	131.78 ± 109.46 (3.41 ± 2.83)	151.24 ± 121.14 (3.91 ± 3.13)	<0.001
** HDL-C, mg/dL (mmol/L)**	43.50 ± 13.50 (1.13 ± 0.35)	43.57 ± 13.50 (1.13 ± 0.35)	42.23 ± 13.30 (1.09 ± 0.34)	0.001	43.23 ± 13.00 (1.12 ± 0.34)	43.93 ± 14.02 (1.14 ± 0.36)	<0.001
** LDL-C, mg/dL (mmol/L)**	112.19 ± 44.08 (2.90 ± 1.14)	112.88 ± 43.87 (2.92 ± 1.13)	98.06 ± 45.85 (2.54 ± 1.19)	<0.001	107.31 ± 38.72 (2.78 ± 1.00)	118.91 ± 48.12 (3.08 ± 1.24)	<0.001
** HbA1c, mg/dL**	6.43 ± 1.42	6.43 ± 1.42	6.56 ± 1.40	0.005	6.42 ± 1.40	6.43 ± 1.44	0.020
**Laboratory results at 1 year**							
** TC, mg/dL (mmol/L)**	134.67 ± 32.22 (3.48 ± 0.83)	134.01 ± 31.54 (3.47 ± 0.82)	151.95 ± 43.04 (3.93 ± 1.11)	<0.001	137.81 ± 31.47 (3.56 ± 0.81)	130.35 ± 31.19 (3.37 ± 0.81)	<0.001
** TG, mg/dL (mmol/L)**	135.89 ± 88.19 (3.51 ± 2.28)	135.54 ± 88.30 (3.51 ± 2.28)	145.41 ± 84.80 (3.76 ± 2.19)	0.018	137.57 ± 89.68 (3.56 ± 2.32)	133.64 ± 86.95 (3.46 ± 2.25)	0.003
** HDL-C, mg/dL (mmol/L)**	45.25 ± 11.99 (1.17 ± 0.31)	45.31 ± 12.00 (1.17 ± 0.31)	43.52 ± 11.65 (1.13 ± 0.30)	0.002	45.02 ± 11.84 (1.16 ± 0.31)	45.58 ± 12.15 (1.18 ± 0.31)	<0.001
** LDL-C, mg/dL (mmol/L)**	71.27 ± 25.74 (1.84 ± 0.67)	70.70 ± 25.20 (1.83 ± 0.65)	86.79 ± 34.04 (2.24 ± 0.88)	<0.001	73.25 ± 26.27 (1.89 ± 0.68)	68.32 ± 23.91 (1.77 ± 0.62)	<0.001
** HbA1c, mg/dL**	6.58 ± 1.28	6.58 ± 1.28	6.73 ± 1.36	0.031	6.59 ± 1.33	6.57 ± 1.23	0.069

Values are presented as percentages (numbers) for categorical values and as means ± standard deviations for continuous values.

BMI, body mass index; CAD, coronary artery disease; CVA, cerebrovascular accident; EMS, emergency medical service; HbA1c, glycated hemoglobin; HDL-C, high-density lipoprotein cholesterol; LDL-C, low-density lipoprotein cholesterol; LVEF, left ventricular ejection fraction; PCI, percutaneous coronary intervention; RAAS, renin-angiotensin-aldosterone system; STEMI, ST-elevation myocardial infarction; TC, total cholesterol; TG, triglyceride.

Overall, the statin group included younger patients, had higher obesity prevalence, and included more men and smokers than the no-statin group. Except for dyslipidemia (including dyslipidemia upon treatment), most comorbidities (medical and family histories of coronary artery disease) were more prevalent in the no-statin group than in the statin group. Moreover, the no-statin group had a higher proportion of Killip functional classes III–IV, more cases of impaired kidney function, and a lower LVEF than did the statin group. However, the statin group had more final diagnoses of STEMI than did the no-statin group. These results were more pronounced with high- than with moderate-intensity statin use.

Regarding treatment, the statin group received more PCIs and prescriptions of aspirin, purinergic P2Y receptor G protein-coupled 12 inhibitors, beta-blockers, and renin-angiotensin-aldosterone system inhibitors than did the no-statin group.

The initial laboratory results suggested that all lipid profile parameters were lower in the no-statin group than in the statin group. However, these trends were reversed at 1 year, except for high-density lipoprotein cholesterol (HDL-C) concentrations. The glycated hemoglobin (HbA1c) concentrations were higher in the statin group than in the no-statin group at the initial stage and 1 year. The laboratory data trends were more pronounced with high- than with moderate-intensity statin use.

According to the data of the LLA-naïve participants (**S1 Text**), the trends for most items persisted, except for HbA1c concentrations at both the initial stage and at 1 year. For this dataset, the differences between the HbA1c concentrations at the initial stage and 1 year were statistically attenuated.

### Temporal trends in dyslipidemia prevalence and drug prescriptions

**Fig 2:**
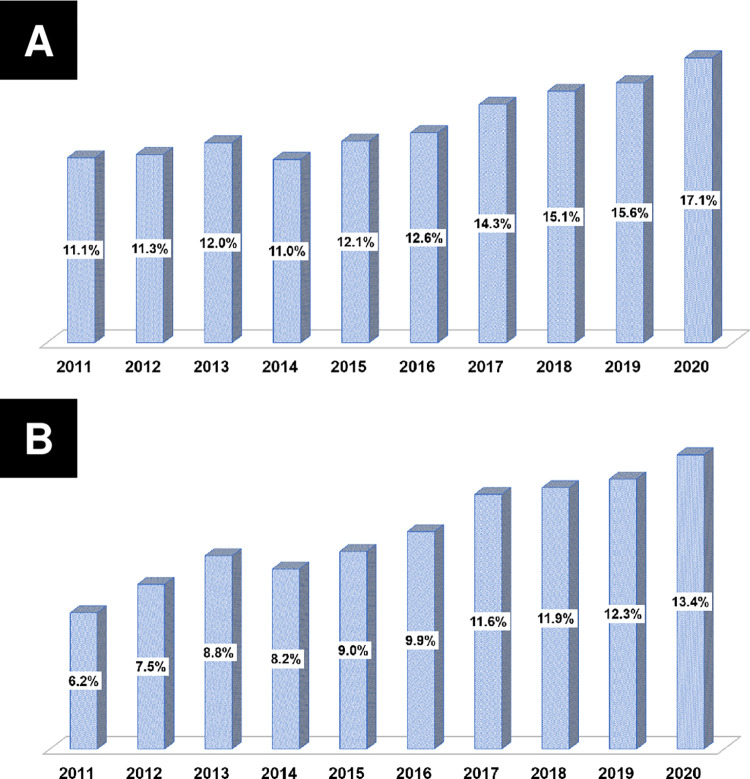
Temporal trends in the prevalence of dyslipidemia. (A) Prevalence of dyslipidemia; (B) Prevalence of dyslipidemia treated with lipid-lowering agents.

**Fig 3:**
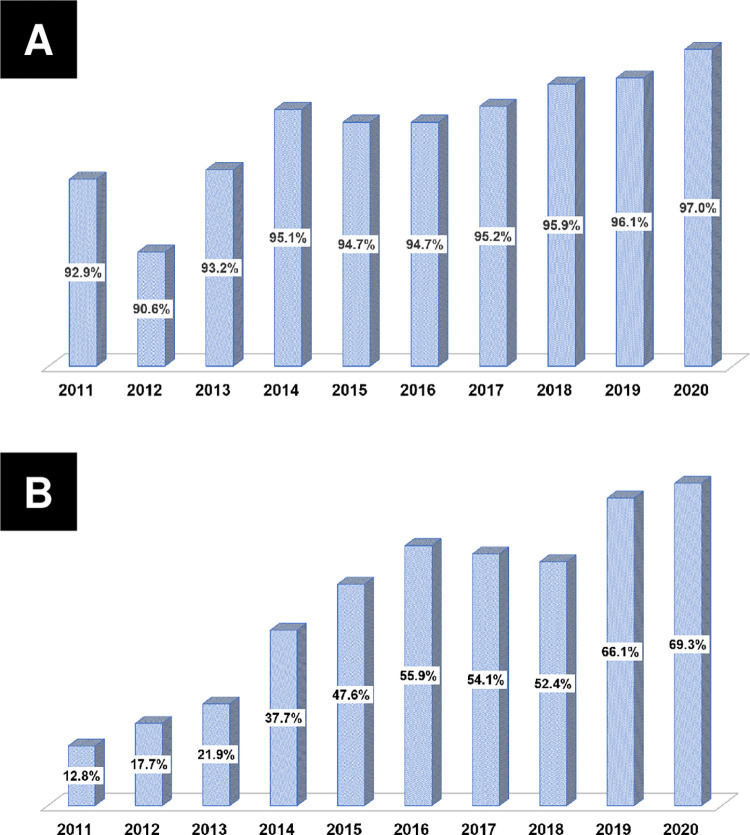
Temporal trends in the prescription fill rates of statins. (A) Statin prescription fill rates; (B) High-intensity statin prescription fill rates.

**Fig 4:**
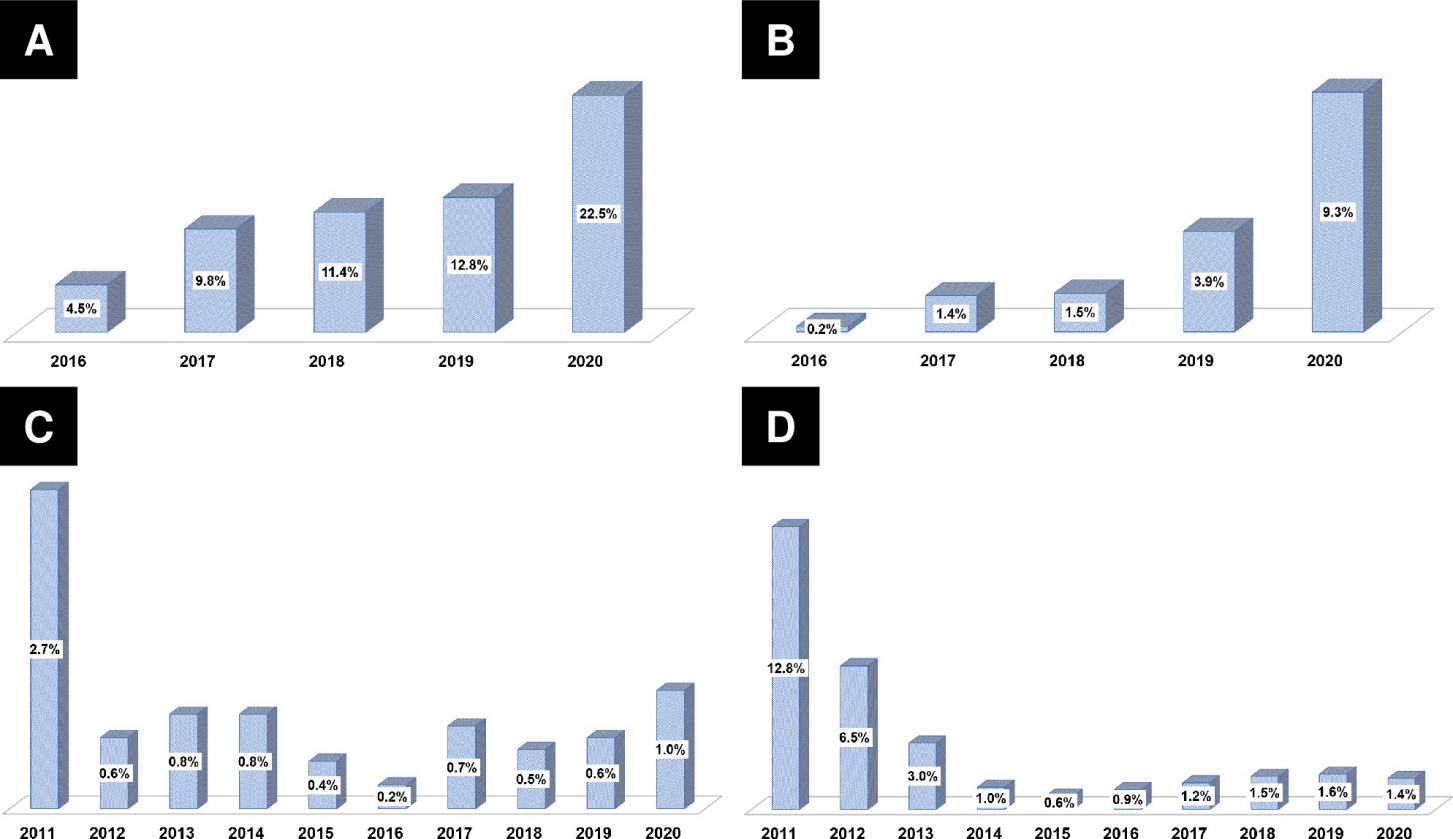
Temporal trends in the prescription fill rates of non-statin lipid-lowering agents. (A) Ezetimibe prescription fill rates; (B) High-intensity statin plus ezetimibe prescription fill rates; (C) Fibrate prescription fill rates; (D) Omega-3 fatty acid prescription fill rates.

**Table 2** summarizes the temporal trends in the prevalence of dyslipidemia and LLA prescriptions. Since 2011, the prevalence of dyslipidemia has increased (all *p*<0.001) (**Fig 2A**). The number of patients receiving LLAs for dyslipidemia has also increased (all *p*<0.001) (**Fig 2B**). The prescription rate of statins showed an increase from 92.90% in 2011 to 97.00% in 2020 (all *p*<0.001) (**Fig 3A**). Furthermore, the prescription rate of high-intensity statins gradually increased from 12.80% in 2012 to 69.30% in 2020 (all *p*<0.001) (**Fig 3B**). In the KAMIR-V registry, the ezetimibe prescription rates demonstrated a gradual increase from 4.50% in 2016 to 22.50% in 2020 (all *p*<0.001) (**Fig 4A**). The prescription rates of high-intensity statins along with ezetimibe demonstrated a similar increase from 0.20% in 2016 to 9.30% in 2020 (all *p*<0.001) (**Fig 4B**). In contrast, the fibrate prescription rates were generally less than 5% (all *p* = 0.273). Similarly, the prescription rates of omega-3 fatty acids were predominantly less than 5%, except from 2011 to 2012 (all *p*<0.001) (**Fig 4C and 4D**).

**Table 2:** Temporal trends in dyslipidemia prevalence and medication prescriptions in the study cohort.

	The KAMIR-NIH registry (2011–2015)					The KAMIR-V registry (2016–2020)					*p* for trend
	2011	2012	2013	2014	2015	2016	2017	2018	2019	2020	
	N = 226	N = 2,992	N = 3,229	N = 3,594	N = 2,428	N = 3,370	N = 3,829	N = 3,448	N = 2,747	N = 888	
**Prevalence**											
**Dyslipidemia**	25 (11.1)	338 (11.3)	388 (12.0)	397 (11.0)	295 (12.1)	425 (12.6)	548 (14.3)	522 (15.1)	428 (15.6)	152 (17.1)	<0.001
**Dyslipidemia on treatment**	14 (6.3)	224 (7.5)	184 (8.8)	294 (8.2)	218 (9.0)	332 (9.9)	446 (11.7)	409 (12.0)	339 (12.5)	119 (13.6)	<0.001
**Prescription fill rates**											
** Statins**	210 (92.9)	2,711 (90.6)	3,010 (93.2)	3,419 (95.1)	2,300 (94.7)	3,191 (94.7)	3,644 (95.2)	3,306 (95.9)	2,640 (96.1)	861 (97.0)	<0.001
** Moderate-intensity statins**	181 (80.1)	2,181 (72.9)	2,304 (71.4)	2,065 (57.5)	1,145 (47.2)	1,308 (38.8)	1,573 (41.1)	1,499 (43.5)	823 (30.0)	246 (27.7)	<0.001
** High-intensity statins**	29 (12.8)	530 (17.7)	706 (21.9)	1,354 (37.7)	1,155 (47.6)	1,883 (55.9)	2,071 (54.1)	1,807 (52.4)	1,817 (66.1)	615 (69.3)	<0.001
** Ezetimibe**	NA	NA	NA	NA	NA	153 (4.5)	375 (9.8)	394 (11.4)	351 (12.8)	200 (22.5)	<0.001
** Fibrates**	6 (2.7)	17 (0.6)	26 (0.8)	28 (0.8)	10 (0.4)	8 (0.2)	25 (0.7)	18 (0.5)	17 (0.6)	9 (1.0)	0.273
** Omega-3 fatty acids**	29 (12.8)	194 (6.5)	97 (3.0)	37 (1.0)	15 (0.6)	32 (0.9)	45 (1.2)	50 (1.5)	43 (1.6)	12 (1.4)	<0.001

Values are presented as percentages (numbers) for categorical values.

KAMIR-NIH, Korea Acute Myocardial Infarction Registry-National Institutes of Health; KAMIR-V, Korea Acute Myocardial Infarction Registry-V; NA, not available.

### Temporal trends in lipid profiles and achievement of LDL-C target goals

**Fig 5:**
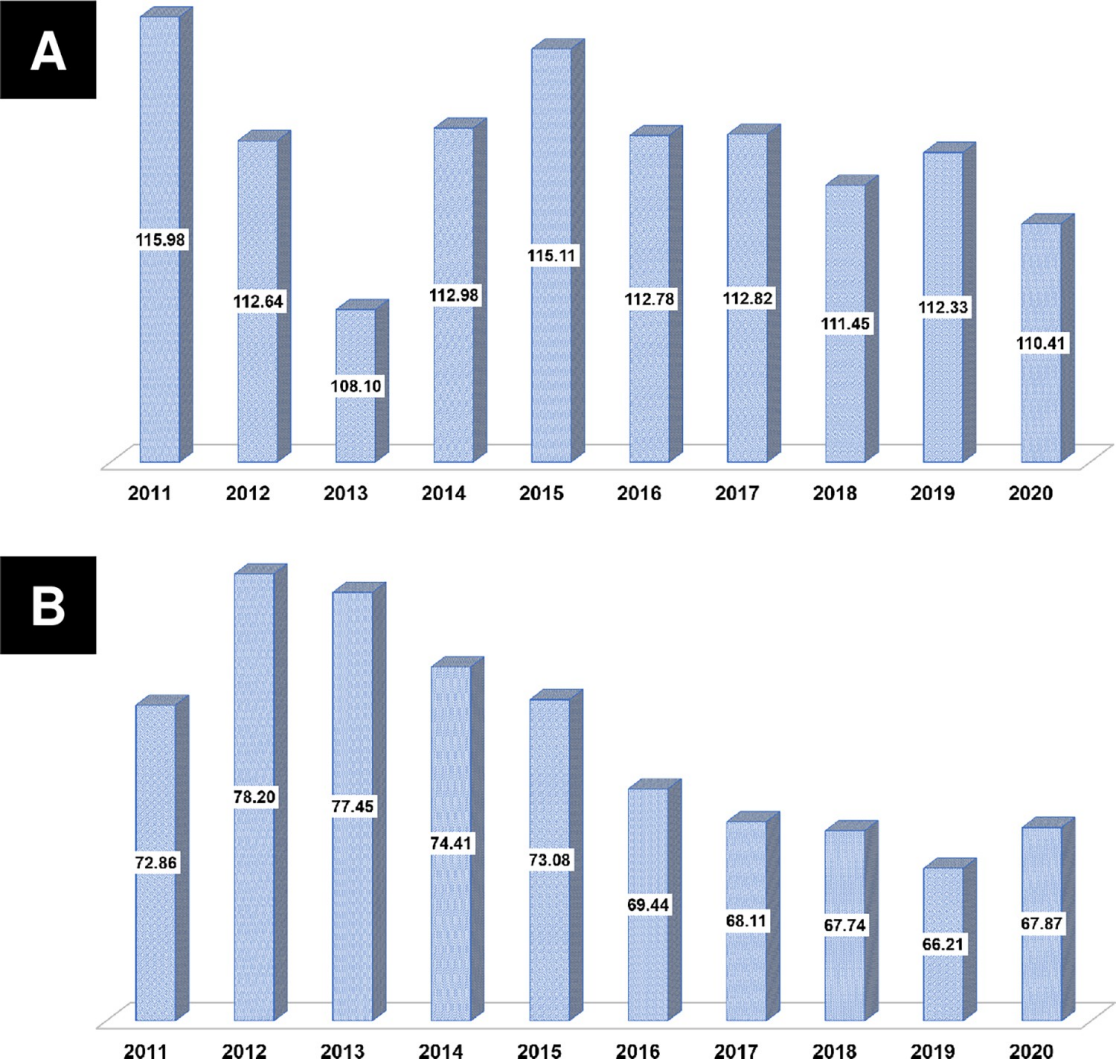
Temporal trends in LDL-C levels during index hospitalization (at baseline) and at 1 year after AMI. AMI, acute myocardial infarction; LDL-C, low-density lipoprotein cholesterol.

**Fig 6:**
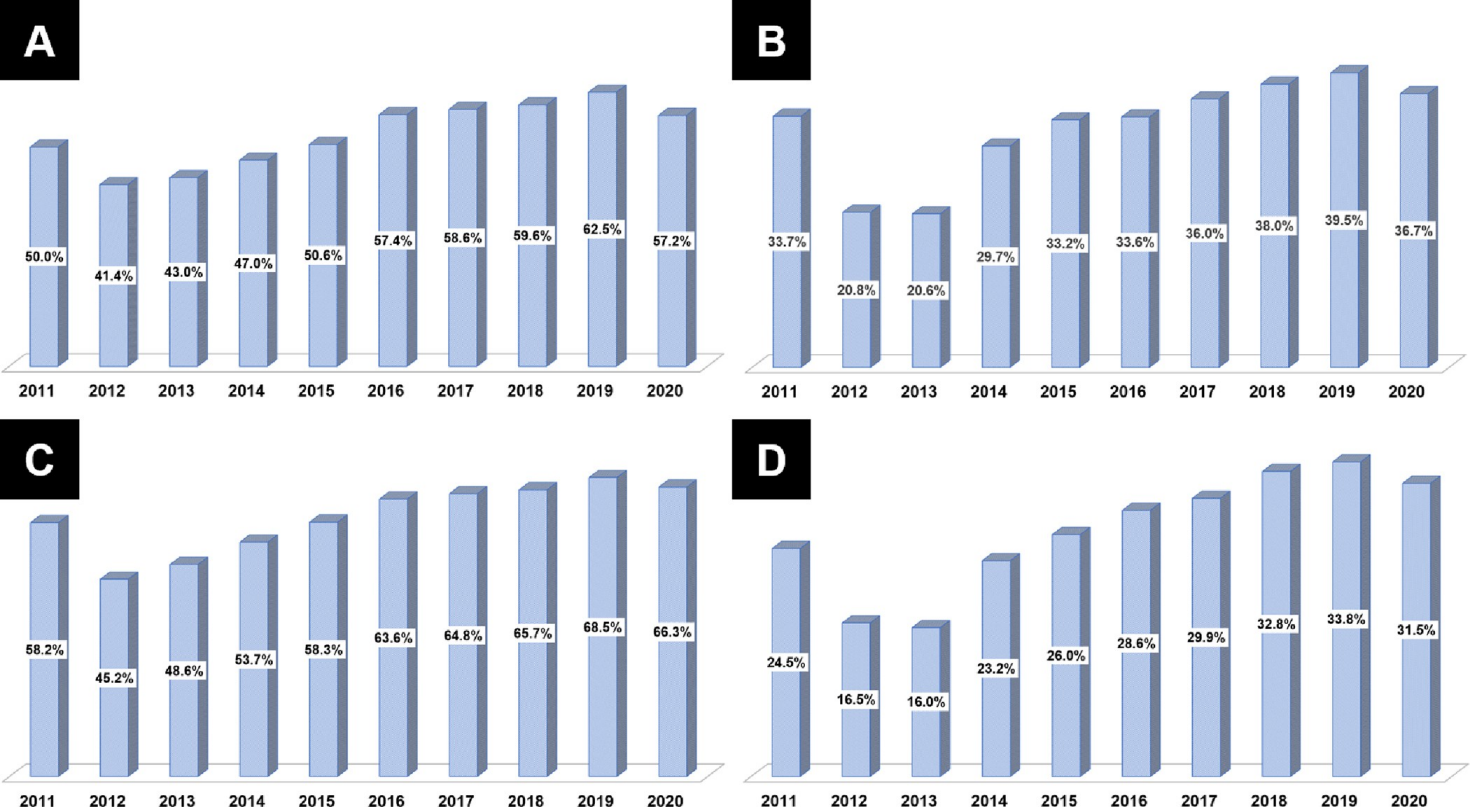
Temporal trends in the attainment rates of LDL-C targets. (A) Absolute goal; (B) Relative goal; (C) The American goal; (D) The European goal. LDL-C, low-density lipoprotein cholesterol.

**Table 3** summarises the temporal trends in lipid profiles and achievement of LDL-C target goals. Using our pooled database, we conducted a temporal trend analysis of LDL-C concentrations during the index hospitalization (at baseline) and at 1 year after AMI (**Fig 5**). The initial mean LDL-C concentrations were >100 mg/dL (>2.6 mmol/L) every year (all *p*<0.001). However, the mean follow-up LDL-C concentrations showed a gradual decline from 78.2 mg/dL (2.02 mmol/L) in 2012 to 66.2 mg/dL (1.71 mmol/L) in 2019 (all *p*<0.001), and these concentrations were consistently maintained at values <70 mg/dL (<1.8 mmol/L) from 2016 to 2020. A temporal trend analysis of the achievement rates of the LDL-C target goal demonstrated similar increasing trends for each primary endpoint (**Fig 6**). The achievement rate of the absolute LDL-C target goal increased from 41.4% in 2012 to 62.5% in 2019 (all *p*<0.001), whereas that of the relative LDL-C target goal increased from 20.8% in 2012 to 39.5% in 2019 (all *p*<0.001). The achievement rates of the American (from 45.7% in 2012 to 68.6% in 2019) (all *p*<0.001) and European (from 16.5% in 2012 to 33.8% in 2019) (all *p*<0.001) goals displayed similar increasing trends.

**Table 3:** Temporal trends in lipid management in the study cohort.

	The KAMIR-NIH registry (2011–2015)					The KAMIR-V registry (2016–2020)					*p* for trend
	2011	2012	2013	2014	2015	2016	2017	2018	2019	2020	
	N = 226	N = 2,992	N = 3,229	N = 3,594	N = 2,428	N = 3,370	N = 3,829	N = 3,448	N = 2,747	N = 888	
**Laboratory results at the initial stage**											
** TC, mg/dL (mmol/L)**	184.38 ± 41.81 (4.77 ± 1.08)	179.15 ± 43.71 (4.63 ± 1.13)	176.65 ± 47.06 (4.57 ± 1.22)	178.59 ± 45.25 (4.62 ± 1.17)	179.80 ± 47.27 (4.65 ± 1.22)	177.52 ± 46.26 (4.59 ± 1.20)	178.11 ± 51.41 (4.61 ± 1.33)	176.46 ± 46.07 (4.56 ± 1.19)	176.08 ± 46.76 (4.55 ± 1.21)	181.93 ± 104.02 (4.71 ± 2.69)	0.009
** TG, mg/dL (mmol/L)**	144.67 ± 108.99 (3.74 ± 2.82)	134.28 ± 114.72 (3.47 ± 2.97)	129.50 ± 117.56 (3.35 ± 3.04)	134.71 ± 99.42 (3.48 ± 2.57)	138.13 ± 130.52 (3.57 ± 3.38)	142.59 ± 108.38 (3.69 ± 2.80)	150.84 ± 116.39 (3.90 ± 3.01)	144.04 ± 109.67 (3.73 ± 2.84)	148.14 ± 125.28 (3.83 ± 3.24)	148.01 ± 123.24 (3.83 ± 3.19)	<0.001
** HDL-C, mg/dL (mmol/L)**	40.94 ± 10.05 (1.06 ± 0.26)	42.90 ± 11.92 (1.11 ± 0.31)	42.30 ± 11.51 (1.09 ± 0.30)	43.35 ± 12.01 (1.12 ± 0.31)	43.43 ± 11.93 (1.12 ± 0.31)	43.92 ± 15.37 (1.14 ± 0.40)	43.74 ± 14.14 (1.13 ± 0.37)	44.16 ± 15.58 (1.14 ± 0.40)	44.25 ± 15.41 (1.14 ± 0.40)	43.50 ± 13.50 (1.13 ± 0.35)	<0.001
** LDL-C, mg/dL (mmol/L)**	115.98 ± 36.61 (3.00 ± 0.95)	112.64 ± 39.56 (2.91 ± 1.02)	108.10 ± 38.36 (2.80 ± 0.99)	112.98 ± 39.38 (2.92 ± 1.02)	115.11 ± 40.57 (2.98 ± 1.05)	112.78 ± 59.04 (2.92 ± 1.53)	112.82 ± 45.02 (2.92 ± 1.16)	111.45 ± 42.23 (2.88 ± 1.09)	112.33 ± 45.94 (2.91 ± 1.19)	110.41 ± 39.27 (2.86 ± 1.02)	<0.001
** HbA1c, mg/dL**	6.92 ± 1.40	6.66 ± 1.53	6.42 ± 1.40	6.41 ± 1.46	6.43 ± 1.46	6.43 ± 1.44	6.42 ± 1.43	6.36 ± 1.30	6.38 ± 1.37	6.43 ± 1.42	<0.001
**Laboratory results at 1 year**											
** TC, mg/dL (mmol/L)**	140.93 ± 28.53 (3.64 ± 0.74)	145.15 ± 31.24 (3.75 ± 0.81)	141.87 ± 33.01 (3.67 ± 0.85)	137.88 ± 32.35 (3.57 ± 0.84)	137.06 ± 38.38 (3.54 ± 0.99)	132.83 ± 31.61 (3.44 ± 0.82)	130.72 ± 28.97 (3.38 ± 0.75)	130.21 ± 31.02 (3.37 ± 0.80)	128.19 ± 29.69 (3.32 ± 0.77)	129.16 ± 29.14 (3.34 ± 0.75)	<0.001
** TG, mg/dL (mmol/L)**	143.72 ± 103.27 (3.72 ± 2.67)	136.78 ± 78.92 (3.54 ± 2.04)	135.38 ± 87.33 (3.50 ± 2.26)	137.75 ± 96.67 (3.56 ± 2.5)	139.37 ± 106.28 (3.60 ± 2.75)	136.18 ± 83.40 (3.52 ± 2.16)	139.21 ± 87.11 (3.60 ± 2.25)	135.00 ± 87.71 (3.49 ± 2.27)	129.25 ± 78.56 (3.34 ± 2.03)	126.77 ± 73.38 (3.28 ± 1.90)	0.016
** HDL-C, mg/dL (mmol/L)**	43.16 ± 11.69 (1.12 ± 0.30)	44.04 ± 10.95 (1.14 ± 0.28)	44.63 ± 12.22 (1.15 ± 0.32)	44.74 ± 11.44 (1.16 ± 0.30)	44.59 ± 11.71 (1.15 ± 0.30)	45.95 ± 12.68 (1.19 ± 0.33)	45.84 ± 12.83 (1.19 ± 0.33)	45.37 ± 11.49 (1.17 ± 0.30)	45.88 ± 11.13 (1.19 ± 0.29)	46.66 ± 14.24 (1.21 ± 0.37)	<0.001
** LDL-C, mg/dL (mmol/L)**	72.86 ± 23.93 (1.88 ± 0.62)	78.20 ± 27.05 (2.02 ± 0.70)	77.45 ± 26.89 (2.00 ± 0.70)	74.41 ± 26.16 (1.92 ± 0.68)	73.08 ± 25.71 (1.89 ± 0.66)	69.44 ± 25.73 (1.80 ± 0.67)	68.11 ± 23.08 (1.76 ± 0.60)	67.74 ± 26.31 (1.75 ± 0.68)	66.21 ± 23.84 (1.71 ± 0.62)	67.87 ± 23.19 (1.76 ± 0.60)	<0.001
** HbA1c, mg/dL**	6.74 ± 1.44	6.65 ± 1.37	6.53 ± 1.29	6.64 ± 1.34	6.60 ± 1.27	6.61 ± 1.33	6.59 ± 1.34	6.51 ± 1.14	6.57 ± 1.26	6.57 ± 1.02	0.331
**LDL-C target goal**											
** Absolute goal**	51 (50.0)	487 (41.4)	579 (43.0)	796 (47.0)	617 (50.6)	968 (57.4)	1,100 (58.6)	1,024 (59.6)	913 (62.5)	275 (57.2)	<0.001
** Relative goal**	33 (33.7)	209 (20.8)	255 (20.6)	476 (29.7)	385 (33.2)	494 (33.6)	599 (36.0)	591 (38.0)	505 (39.5)	135 (36.7)	<0.001
** American goal**	57 (58.2)	460 (45.7)	601 (48.6)	860 (53.7)	675 (58.3)	936 (63.6)	1,079 (64.8)	1,023 (65.7)	877 (68.5)	244 (66.3)	<0.001
** European goal**	24 (24.5)	166 (16.5)	198 (16.0)	371 (23.2)	301 (26.0)	420 (28.6)	498 (29.9)	510 (32.8)	433 (33.8)	116 (31.5)	<0.001

Values are presented as percentages (numbers) for categorical values and as means ± standard deviations for continuous values.

HbA1c, glycated hemoglobin; HDL-C, high-density lipoprotein cholesterol; KAMIR-NIH, Korea Acute Myocardial Infarction Registry-National Institutes of Health; KAMIR-V, Korea Acute Myocardial Infarction Registry-V; LDL-C, low-density lipoprotein cholesterol; TC, total cholesterol; TG, triglyceride.

For the LLA-naïve participants (**S2 Text**), all trends were maintained at levels similar to those for the overall study cohort, with the exception of the triglyceride (TG) trend at 1 year.

### Association between statin intensity and achievement of LDL-C target goals

**Table 4** summarizes the correlation between statins, their intensity, and achievement of LDL-C target goals using different models. The LDL-C target goals were achieved with high-intensity statins approximately 2.49–2.99 times more than with no statins. The statin group achieved LDL-C target goals approximately 1.33–1.93 times more than the no-statin group.

**Table 4:** Associations between statin strategies and the incidence of LDL-C target goal attainment according to each Cox model.

	Statin strategies				Model 1	Model 2	Model 3	Model 4
	No statin (N = 459)	Moderate-intensity statins (N = 5,929)	High-intensity statins (N = 6,373)		HR (95% CI)	HR (95% CI)	HR (95% CI)	HR (95% CI)
**Absolute LDL-C target goal**	167 (36.4)	2,901 (48.9)	3,742 (58.7)	Moderate-intensity statins	1.68 (1.38–2.04)	1.68 (1.38–2.05)	1.91 (1.53–2.37)	1.93 (1.54–2.42)
				High-intensity statins	2.49 (2.04–3.03)	2.50 (2.05–3.04)	2.88 (2.32–3.59)	2.97 (2.37–3.72)
**Relative LDL-C target goal**	71 (19.2)	1,277 (24.1)	2,334 (40.4)	Moderate-intensity statins	1.34 (1.02–1.74)	1.33 (1.02–1.74)	1.28 (0.95–1.72)	1.20 (0.89–1.62)
				High-intensity statins	2.84 (2.18–3.70)	2.80 (2.15–3.65)	2.66 (1.99–3.57)	2.52 (1.87–3.40)
**American goal**	163 (44.2)	2,808 (53.1)	3,841 (66.4)	Moderate-intensity statins	1.43 (1.16–1.77)	1.43 (1.16–1.77)	1.54 (1.22–1.94)	1.53 (1.21–1.95)
				High-intensity statins	2.50 (2.02–3.09)	2.50 (2.02–3.09)	2.71 (2.15–3.42)	2.73 (2.15–3.46)
**European goal**	52 (14.1)	1,080 (20.4)	1,905 (32.9)	Moderate-intensity statins	1.56 (1.16–2.11)	1.56 (1.16–2.11)	1.54 (1.10–2.14)	1.45 (1.03–2.03)
				High-intensity statins	2.99 (2.22–4.04)	2.96 (2.19–3.99)	2.90 (2.08–4.03)	2.78 (1.99–3.89)

Model 1: Crude model.

Model 2: Adjusted for age and sex.

Model 3: Adjusted for all components in Model 2, plus use of EMS, Killip functional class, BMI, smoking status, past medical history, family history of CAD, and serum creatinine level.

Model 4: Adjusted for all components in Model 3 plus post-discharge medications, LVEF, PCI utilization, and final diagnosis.

BMI, body mass index; CAD, coronary artery disease; CI, confidence interval; EMS, emergency medical service; HR, hazard ratio; LDL-C, low-density lipoprotein cholesterol; LVEF, left ventricular ejection fraction; PCI, percutaneous coronary intervention.

An identical statistical analysis of the data of the LLA-naïve participants (**S3 Text**) showed trends equivalent to those of the overall study cohort. The LDL-C target goals were achieved with the high-intensity statins approximately 2.75–3.28 times more than without statins. Additionally, the LDL-C target goals were achieved with moderate-intensity statins approximately 1.32–2.13 times more than with the reference group.

## Discussion

### Main findings

Using the pooled data of two continuous, large-scale, nationwide, multicenter cohorts (the KAMIR-NIH and KAMIR-V registries), we examined the baseline characteristics of patients with AMI and temporal trends in lipid management in the Republic of Korea.

The statin group included younger patients, had higher obesity prevalence, and included more men and smokers than the no-statin group. The higher proportion of men and smokers in the statin group can be attributed to the cumulative evidence of the predominance of tobacco smoking in men [18]. Moreover, smoking habits may sustain an earlier first-onset AMI [19], whereas obesity facilitates increased LDL-C concentrations [20], suggesting that the observed distributions are sufficiently reasonable. Additionally, the statin group demonstrated a lower Killip functional class grade, better kidney and heart function, and higher LVEF than did the no-statin group. The statin group also had more final diagnoses of STEMI than did the no-statin group. Considering that these variables are well-established independent predictors of adverse events in this population [21–24], statins appear to be prescribed more often in clinically healthier patients. According to our stratified analysis, most of the distribution trends appeared to be amplified with increased statin intensity.

**Fig 7:**
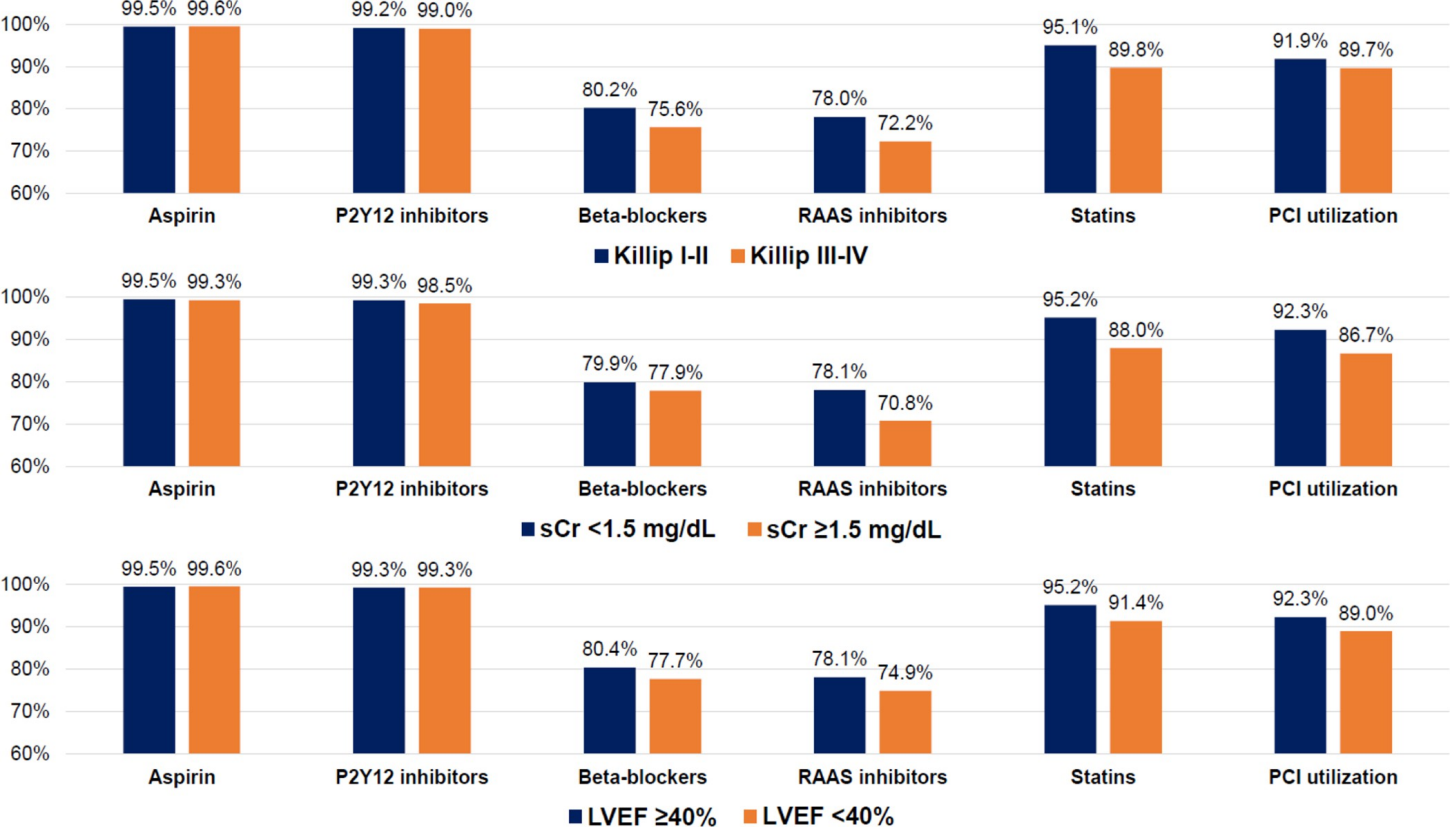
Proportions of prescriptions of post-discharge medications and PCI utilization according to Killip functional class, serum creatinine levels, and LVEF. LVEF, left ventricular ejection fraction; PCI, percutaneous coronary intervention; RAAS, renin-angiotensin-aldosterone system; sCr, serum creatinine.

Further investigation (**S4 Text and Fig 7**) revealed that clinically healthier patients, such as those with lower Killip functional class (Killip I–II), lower serum creatinine concentrations (<1.5 mg/dL), or higher LVEF (≥40%), tend to receive more optimal pharmacological and interventional treatments. These subgroups had more prescriptions of beta-blockers, renin-angiotensin-aldosterone system inhibitors, and statins and higher PCI use than did their counterparts. These drugs and rapid reperfusion therapy are well-established treatments for AMI [4,25], and they are usually administered in real-world practice. A clinical study of nonagenarian patients with AMI suggested that PCI-treated patients received more optimal pharmacotherapy than their non-PCI-treated counterparts [26]. These findings are consistent with our results. However, further research is required to confirm them.

In the temporal trend analysis, the prevalence of dyslipidemia and LLA prescription rates progressively increased. The incremental trends in statin prescription may provide substantial evidence that statins targeting LDL-C significantly reduce major adverse cardiovascular events [27,28]. The yearlong increases in the prescription rates of high-intensity statins were prominent between 2013 (21.9%) and 2014 (37.7%) and between 2018 (52.4%) and 2019 (66.1%) (**Fig 3B**). This may have been influenced by major revisions to the international lipid management guidelines [29,30]. In 2013, the Joint ACC/AHA Task Force on Practice Guidelines emphasized the use of high-intensity statins for secondary prevention in patients with established ASCVDs [30], indicating that the major focus of lipid management had shifted from prioritizing absolute LDL-C values to highlighting the intensity of statins [31]. In both the 2016 ESC/EAS guidelines and the updated 2018 ACC/AHA guidelines, the LDL-C threshold was defined as 70 mg/dL (1.8 mmol/L) for patients with high-risk ASCVD. Patients with values above this threshold were recommended high-intensity statin use or maximally tolerated statin therapy [29].

Statins are increasingly available and are strongly recommended for AMI management due to their impressive LDL-C-lowering efficacy. Nonetheless, some patients with minimal risks of ASCVDs still require LDL-C reduction [32]. Ezetimibe is one of the non-statin agents for reducing the concentration of LDL-C [33]. It blocks the Niemann-Pick C1-like transporter at the jejunal enterocyte brush border and hinders LDL-C uptake [34]. The IMPROVE-IT trial reported its clinical benefit of major ASCVD risk reduction [35]; thus, the 2018 ACC/AHA guidelines recommended the addition of ezetimibe to maximally tolerated statins if the LDL-C target is not achieved [29]. Our results, which suggest the progressively increasing prescription rates of ezetimibe, may reflect the increased adoption of clinical guidelines in real-world practice.

Thanks to the improved prescription of LLAs highlighted above, LDL-C concentrations at 1 year decreased more prominently in the statin group than in the no-statin group, showing an overall year-over-year declining trend. Moreover, the achievement rates of all components of the LDL-C goals increased gradually. This improvement in LDL-C management may reflect the increased adoption of the clinical guidelines in real-world practice [16,29].

While the present study defined the absolute LDL-C target goal as 70 mg/dL (1.8 mmol/L), the latest guidelines set a new goal for the LDL-C level. The 2019 ESC/EAS cholesterol guidelines recommended a lower LDL-C target goal (55 mg/dL, 1.4 mmol/L) for a more aggressive LDL-C reduction in the AMI population [10]. The 2022 Korean Guidelines for the Management of Dyslipidemia also endorsed this new target goal, heightening LDL-C reduction efforts to optimize lipid management [36]. Although most participants included in the present study were enrolled before these new guidelines were published, our further trend analysis showed a slight but gradual increase in the proportion of patients who attained these more stringent LDL-C levels (**S5 Text**). These trends may also be influenced by multiple lines of evidence supporting the cardiovascular benefits of intensive LDL-C reduction [13,35].

Despite these gradual improvements, the inability of patients to decrease their LDL-C concentrations to target goals should be considered. More than 50% of patients with AMI fail to achieve the European goal (**Fig 7**). These unsatisfactory results imply that achieving the LDL-C goal has remained suboptimal in real-world practice, and the gap between the guidelines and clinical practice for treating dyslipidemia in this population has remained unaddressed. These real-world unmet needs may be consistent with the results of previous cohort studies in the literature [37–41]. In a Spanish cohort study, more than half of patients receiving high-intensity statins failed to achieve the LDL-C goals for primary or secondary prevention [40]. In a Turkish nationwide registry, most of the familial hypercholesterolemia patients with established ASCVD did not receive high-intensity statin therapy, and they failed to achieve the proposed LDL-C target goals [41].

Although speculative but still not fully accountable, these undertreatments may be influenced not only by patient-related issues such as side effects or non-preferences but also by the therapeutic inertia of cardiologists and their knowledge gaps. A clinical study of veterans in the United States reported that more than half of veterans did not receive LLA intensification, and clinical processes such as outpatient lipid testing or visiting a cardiologist resulted in higher LLA intensification [42], which suggests the importance of physicians’ therapeutic inertia. Moreover, Korean survey data demonstrated that many Korean clinicians do not agree with lowering the LDL-C levels to <55 mg/dL because its safety and efficacy have not yet been proven in ethnic Koreans [43]. This survey reflects a substantial disparity between current guidelines and real-world practices, highlighting the necessity for further investigations to bridge this gap.

As the use of statins is independently associated with a higher likelihood of achieving the LDL-C target goals (**Table 4 and Fig 7 and S3 Text**), multifaceted and continued efforts for increased statin use are warranted to optimize lipid management and reduce the risk of recurrent ischemic events. If the maximally tolerated dose of statin is insufficient to optimally reduce LDL-C levels, clinicians should consider the combination therapy with novel LLAs such as ezetimibe or proprotein convertase subtilisin/kexin type 9 inhibitors.

### Strengths and weaknesses of the study

The major strength of our study was the inclusion of participants with AMI in the Republic of Korea through two nationwide observational cohorts. Our novel epidemiological investigation highlights the trends in lipid management among patients with AMI living in the Republic of Korea, one of the East Asian nations. To our best knowledge, our analysis is the first to assess the temporal trends in lipid management in an East Asian ethnic group.

Nonetheless, this study had some limitations. First, the two nationwide observational cohorts only had clinical data from high-volume tertiary medical institutions. Therefore, they do not reflect the multifaceted trends in primary healthcare centers across the Republic of Korea. Second, some detailed information was not available in this study, and this may have contributed to residual confounding factors. We did not provide any information about LLAs, including their duration of use, switching, or discontinuation. Moreover, we did not provide any information on novel therapeutics, such as proprotein convertase subtilisin/kexin type 9 inhibitors or bempedoic acid, which are paving the way to a novel chapter in the treatment of dyslipidemia. We did not include any information on the prevalence of familial hypercholesterolemia. Third, the prevalence of dyslipidemia may have been underestimated because the past medical records were predominantly based on the self-reported data of the participants [44].

## Conclusions

An increase in the adoption of lipid management guidelines has been observed in real-world practice for the management of Korean patients with AMI. Nonetheless, some patients are unable to decrease their LDL-C concentrations to the target goals. Our real-world analyses emphasize the need for multidirectional and continued efforts to optimize lipid management and improve the clinical outcomes of these patients.

## Supporting information

S1 Text Baseline characteristics of the participants in the study cohort, excluding those with dyslipidemia on treatment (LLA-naïve participants). (PDF)

S2 Text Temporal trends in lipid management in the study cohort excluding those with dyslipidemia on treatment (LLA-naïve participants). (PDF)

S3 Text Associations between statin strategies and the incidence of LDL-C target goal attainment according to each Cox model among LLA-naïve participants. (PDF)

S4 Text Proportions of prescriptions of post-discharge medications and PCI utilization according to Killip functional class, serum creatinine levels, and LVEF. (PDF)

S5 Text Temporal trends in lipid management in the study cohort (LDL <55 mg/dL, and LDL <55 mg/dL with relative goal). (PDF)

S1 Graphical abstract (PDF)

## Abbreviations

ACC/AHA: American College of Cardiology/American Heart Association
AMI: acute myocardial infarction
ASCVD: atherosclerotic cardiovascular disease
CI: confidence interval
ESC/EAS: European Society of Cardiology/European Atherosclerosis Society
HbA1c: glycated hemoglobin
HDL-C: high-density lipoprotein cholesterol
HR: hazard ratio
KAMIR: Korean Acute Myocardial Infarction Registry
KAMIR-NIH: Korean Acute Myocardial Infarction Registry-National Institutes of Health
LDL-C: low-density lipoprotein cholesterol
LLA: lipid-lowering agent
LVEF: left ventricular ejection fraction
PCI: percutaneous coronary intervention
STEMI: ST-elevation myocardial infarction
TG: triglyceride
