# Supplementary material for: Ten-year trends in lipid management among patients after myocardial infarction in South Korea
Source: PLoS One. 2024 Oct 3;19(10):e0304710. doi: 10.1371/journal.pone.0304710 (PMC11449489; doi:10.1371/journal.pone.0304710)
Supplement: S1 Text — (PDF) [file pone.0304710.s001.pdf]

S1 Text. Baseline characteristics of the participants in the study cohort excluding those with dyslipidemia on treatment (LLA-naïve participants).

	**Study participants**	**Prescription fills of statins**			**Patterns of statin prescriptions stratified by statin intensity**		
	**(N = 26,751)**	**Statin group (N = 22,733)**	**No statin group (N = 1,339)**	**p-value between two groups**	**Moderate-intensity statins (N = 11,998)**	**High-intensity statins (N = 10,735)**	**p-value between three groups**
Demographics							
Age ≥75 years	5,434 (22.6)	5,029 (22.1)	405 (30.2)	<0.001	3,040 (25.3)	1,989 (18.5)	<0.001
Age, years	63.58 ± 12.55	63.43 ± 12.52	66.12 ± 12.88	<0.001	64.60 ± 12.58	62.13 ± 12.33	<0.001
Male sex	18,445 (76.6)	17,502 (77.0)	943 (70.4)	<0.001	8,908 (74.2)	8,594 (80.1)	<0.001
Smoking history	13,645 (58.4)	13,018 (59.0)	627 (48.9)	<0.001	6,591 (56.5)	6,427 (61.7)	<0.001
Use of EMS	4,155 (17.3)	3,940 (17.3)	215 (16.1)	0.230	2,025 (16.9)	1,915 (17.9)	0.024
BMI, kg/m^2	24.14 ± 3.34	24.16 ± 3.33	23.76 ± 3.51	<0.001	23.93 ± 3.33	24.42 ± 3.31	<0.001
BMI ≥25 kg/m^2	8,218 (36.2)	7,828 (36.4)	390 (32.6)	0.008	3,786 (33.5)	4,042 (39.6)	<0.001

Killip class III-IV	2,403 (10.0)	2,150 (9.5)	253 (18.9)	<0.001	1,233 (10.3)	917 (8.6)	<0.001
Past medical history							
Hypertension	11,453 (47.6)	10,741 (47.2)	712 (53.2)	<0.001	5,980 (49.8)	4,761 (44.4)	<0.001
Diabetes mellitus	6,270 (26.0)	5,792 (25.5)	478 (35.7)	<0.001	3,245 (27.0)	2,547 (23.7)	<0.001
Dyslipidemia	839 (3.5)	806 (3.5)	33 (2.5)	0.036	365 (3.0)	441 (4.1)	<0.001
Dyslipidemia on treatment	N/A	N/A	N/A	N/A	N/A	N/A	N/A
Prior CAD	3,168 (13.2)	2,908 (12.8)	260 (19.4)	<0.001	1,798 (15.0)	1,110 (10.3)	<0.001
Prior CVA	1,437 (6.0)	1,326 (5.9)	111 (8.4)	<0.001	791 (6.6)	535 (5.0)	<0.001
Family history of CAD	1,696 (7.3)	1,630 (7.4)	66 (5.1)	0.002	724 (6.2)	906 (8.8)	<0.001
Serum creatinine ≥1.5 mg/dL	2,240 (9.3)	1,962 (8.6)	278 (20.8)	<0.001	1,182 (9.9)	780 (7.3)	<0.001
Serum creatinine, mg/dL	1.13± 1.73	1.11 ± 1.73	1.45 ± 1.65	<0.001	1.13 ±1.35	1.08 ± 2.08	<0.001
LVEF <40%	2,739 (11.7)	2,497 (11.3)	242 (19.4)	<0.001	1,395 (12.0)	1,102 (10.5)	<0.001
LVEF, %	52.38 ± 10.95	52.51 ± 10.84	50.11 ± 12.62	<0.001	52.09 ± 11.04	52.98 ± 10.59	<0.001
STEMI as a final diagnosis	11,569 (48.1)	11,057 (48.6)	512 (38.2)	<0.001	5,631 (46.9)	5,426 (50.5)	<0.001
PCI utilization	22,089 (91.8)	21,154 (93.1)	935 (69.8)	<0.001	10,884 (90.7)	10,270 (95.7)	<0.001

Post-discharge medications							
Aspirin	23,946 (99.5)	22,657 (99.7)	1,289 (96.3)	<0.001	11,948 (99.6)	10,709 (99.8)	<0.001
P2Y12 inhibitors	23,871 (99.2)	22,600 (99.4)	1,271 (94.9)	<0.001	11,903 (99.2)	10,697 (99.6)	<0.001
Beta-blockers	19,215 (79.8)	18,382 (80.9)	833 (62.2)	<0.001	9,678 (80.7)	8,704 (81.1)	<0.001
RAAS inhibitors	18,711 (77.7)	17,911 (78.8)	800 (59.7)	<0.001	9,536 (79.5)	8,375 (78.0)	<0.001
Fibrates	127 (0.5)	106 (0.5)	21 (1.6)	<0.001	56 (0.5)	50 (0.5)	<0.001
Omega-3 fatty acids	481 (2.0)	460 (2.0)	21 (1.6)	0.247	257 (2.1)	203 (1.9)	0.206
Laboratory results at initial stage							
TC, mg/dL (or mmol/L)	180.11 ± 49.43 (4.66 ± 1.28)	180.99 ± 49.41 (4.68 ± 1.28)	164.27 ± 47.14 (4.25 ± 1.22)	<0.001	175.97 ± 51.52 (4.55 ± 1.33)	186.88 ± 46.13 (4.83 ± 1.19)	<0.001
TG, mg/dL (or mmol/L)	140.40 ± 116.85 (3.63 ± 3.02)	140.98 ± 117.44 (3.65 ± 3.04)	129.55 ± 104.70 (3.35 ± 2.71)	0.001	131.35 ± 110.49 (3.40 ± 2.86)	151.47 ± 123.73 (3.92 ± 3.20)	<0.001
HDL-C, mg/dL (or mmol/L)	43.45 ± 13.62 (1.12 ± 0.35)	43.51 ± 13.62 (1.13 ± 0.35)	42.30 ± 13.55 (1.09 ± 0.35)	0.005	43.16 ± 13.12 (1.12 ± 0.34)	43.89 ± 14.14 (1.14 ± 0.37)	<0.001

LDL-C, mg/dL (or mmol/L)	114.38 ± 44.35 (2.96 ± 1.15)	115.15 ± 44.14 (2.98 ± 1.14)	98.94 ± 45.75 (2.56 ± 1.18)	<0.001	109.26 ± 38.69 (2.83 ± 1.00)	121.54 ± 48.59 (3.14 ± 1.26)	<0.001
HbA1c, mg/dL	6.41 ± 1.42	6.40 ± 1.42	6.52 ± 1.38	0.018	6.40 ± 1.40	6.41 ± 1.45	0.051
Laboratory results at 1 year							
TC, mg/dL (or mmol/L)	134.05 ± 32.15 (3.47 ± 0.83)	133.31 ± 31.41 (3.45 ± 0.81)	153.24 ± 43.38 (3.96 ± 1.12)	<0.001	137.12 ± 31.21 (3.55 ± 0.81)	129.68 ± 31.18 (3.35 ± 0.81)	<0.001
TG, mg/dL (or mmol/L)	134.61 ± 88.38 (3.48 ± 2.29)	134.20 ± 88.40 (3.47 ± 2.29)	146.01 ± 87.36 (3.78 ± 2.26)	0.008	135.96 ± 89.69 (3.52 ± 2.32)	132.55 ± 87.15 (3.43 ± 2.25)	0.004
HDL-C, mg/dL (or mmol/L)	45.25 ± 12.04 (1.17 ± 0.31)	45.31 ± 12.05 (1.17 ± 0.31)	43.59 ± 11.65 (1.13 ± 0.30)	0.005	45.02 ± 11.89 (1.16 ± 0.31)	45.58 ± 12.19 (1.18 ± 0.32)	0.001
LDL-C, mg/dL (or mmol/L)	70.87 ± 25.27 (1.83 ± 0.65)	70.24 ± 24.67 (1.82 ± 0.64)	87.95 ± 33.90 (2.27 ± 0.88)	<0.001	72.77 ± 25.28 (1.88 ± 0.65)	67.91 ± 23.86 (1.76 ± 0.62)	<0.001
HbA1c, mg/dL	6.55 ± 1.27	6.54 ± 1.26	6.69 ±1.36	0.061	6.55 ± 1.31	6.54 ± 1.22	0.173

Values are presented as percentages (numbers) for categorical values and as means ± standard deviations for continuous values.

BMI, body mass index; CAD, coronary artery disease; CVA, cerebrovascular accident; EMS, emergency medical service; HbA1c, glycated hemoglobin; HDL-C, high-density lipoprotein cholesterol; LDL-C, low-density lipoprotein cholesterol; LLA, lipid-lowering agents; LVEF, left

ventricular ejection fraction; RAAS, renin-angiotensin-aldosterone system; STEMI, ST-elevation myocardial infarction; TC, total cholesterol; TG, triglyceride.
