# Supplementary material for: Ten-year trends in lipid management among patients after myocardial infarction in South Korea
Source: PLoS One. 2024 Oct 3;19(10):e0304710. doi: 10.1371/journal.pone.0304710 (PMC11449489; doi:10.1371/journal.pone.0304710)
Supplement: S2 Text — (PDF) [file pone.0304710.s002.pdf]

S2 Text. Temporal trends in lipid management in the study cohort excluding those with dyslipidemia on treatment (LLA-naïve participants).

	**The KAMIR-NIH registry (2011 – 2015)**					**The KAMIR-V registry (2016 – 2020)**					***P* for trend**
	**2011**	**2012**	**2013**	**2014**	**2015**	**2016**	**2017**	**2018**	**2019**	**2020**	
	**N=93**	**N=1,070**	**N=1,196**	**N=1,528**	**N=1,094**	**N=1,476**	**N=1,654**	**N=1,485**	**N=1,260**	**N=406**	
Laboratory results at initial stage											
TC, mg/dL (or mmol/L)	183.90 ± 40.91 (4.76 ± 1.06)	180.34 ± 43.67 (4.66 ± 1.13)	178.23 ± 47.38 (4.61 ± 1.23)	179.77 ± 45.12 (4.65 ± 1.17)	181.21 ± 47.20 (4.69 ± 1.22)	180.07 ± 46.20 (4.66 ± 1.19)	181.36 ± 52.18 (4.69 ± 1.35)	178.96 ± 45.64 (4.63 ± 1.18)	179.11 ± 46.81 (4.63 ± 1.21)	187.15 ± 110.07 (4.84 ± 2.85)	0.009
TG, mg/dL (or mmol/L)	142.10 ± 107.62 (3.68 ± 2.78)	133.57 ± 116.08 (3.45 ± 3.00)	129.38 ± 118.93 (3.35 ± 3.08)	134.55 ± 100.75 (3.48 ± 2.61)	137.44 ± 130.98 (3.55 ± 3.39)	143.36 ± 110.73 (3.71 ± 2.86)	151.74 ± 119.42 (3.92 ± 3.09)	143.97 ± 111.26 (3.72 ± 2.88)	148.44 ± 129.35 (3.84 ± 3.35)	147.77 ± 116.85 (3.82 ± 3.02)	<0.001
HDL-C, mg/dL (or mmol/L)	40.66 ± 10.20 (1.05 ± 0.26)	42.84 ± 12.00 (1.11 ± 0.31)	42.23 ± 11.50 (1.09 ± 0.30)	43.30 ± 12.11 (1.12 ± 0.31)	43.42 ± 11.90 (1.12 ± 0.31)	43.90 ± 15.63 (1.14 ± 0.40)	43.74 ± 14.44 (1.13 ± 0.37)	44.07 ± 15.53 (1.14 ± 0.40)	44.19 ± 15.93 (1.14 ± 0.41)	45.03 ± 11.58 (1.16 ± 0.30)	<0.001
LDL-C, mg/dL (or mmol/L)	115.80 ± 36.51 (2.99 ± 0.94)	113.92 ± 39.54 (2.95 ± 1.02)	109.80 ± 38.43 (2.84 ± 0.99)	114.26 ± 39.17 (2.96 ± 1.01)	116.76 ± 40.34 (3.02 ± 1.04)	115.58 ± 60.57 (2.99 ± 1.57)	116.18 ± 45.51 (3.00 ± 1.18)	113.84 ± 42.22 (2.94 ± 1.09)	115.21 ± 46.53 (2.98 ± 1.20)	114.05 ± 38.14 (2.95 ± 0.99)	<0.001
HbA1c, mg/dL	6.97 ± 1.44	6.66 ± 1.54	6.39 ± 1.38	6.38 ± 1.45	6.42 ± 1.49	6.41 ± 1.44	6.38 ± 1.41	6.33 ± 1.30	6.36 ± 1.39	6.43 ± 1.41	<0.001
Laboratory results at 1 year											
TC, mg/dL (or mmol/L)	139.56 ± 28.00	144.05 ± 30.71	141.09 ± 32.58	136.89 ± 32.27	135.88 ± 38.79	132.66 ± 32.01	130.27 ± 28.85	129.40 ± 30.89	147.77 ± 29.59	128.55 ± 28.79	<0.001

	(3.61 ± 0.72)	(3.73 ± 0.79)	(3.65 ± 0.84)	(3.54 ± 0.83)	(3.51 ± 1.00)	(3.43 ± 0.83)	(3.37 ± 0.75)	(3.35 ± 0.80)	(3.82 ± 0.77)	(3.32 ± 0.74)	
TG, mg/dL (or mmol/L)	137.68 ± 102.16 (3.56 ± 2.64)	134.76 ± 78.35 (3.49 ± 2.03)	133.54 ± 86.30 (3.45 ± 2.23)	135.43 ± 95.53 (3.50 ± 2.47)	138.87 ± 109.05 (3.59 ± 2.82)	135.52 ± 83.93 (3.50 ± 2.17)	137.69 ± 84.11 (3.56 ± 2.18)	133.35 ± 89.03 (3.45 ± 2.30)	128.51 ± 80.98 (3.32 ± 2.09)	128.92 ± 75.90 (3.33 ± 1.96)	0.142
HDL-C, mg/dL (or mmol/L)	43.14 ± 11.89 (1.12 ± 0.31)	43.87 ± 10.83 (1.13 ± 0.28)	44.76 ± 12.19 (1.16 ± 0.32)	44.67 ± 11.55 (1.16 ± 0.30)	44.46 ± 11.69 (1.15 ± 0.30)	46.12 ± 11.69 (1.19 ± 0.30)	45.90 ± 12.97 (1.19 ± 0.34)	45.46 ± 11.83 (1.18 ± 0.31)	45.90 ± 11.07 (1.19 ± 0.29)	46.56 ± 14.59 (1.20 ± 0.38)	<0.001
LDL-C, mg/dL (or mmol/L)	72.60 ± 24.55 (1.88 ± 0.63)	77.61 ± 26.86 (2.01 ± 0.69)	76.87 ± 26.37 (1.99 ± 0.68)	73.94 ± 26.19 (1.91 ± 0.68)	72.09 ± 25.20 (1.86 ± 0.65)	69.46 ± 26.15 (1.80 ± 0.68)	67.96 ± 22.96 (1.76 ± 0.59)	66.75 ± 23.02 (1.73 ± 0.60)	65.92 ± 24.03 (1.70 ± 0.62)	67.71 ± 22.89 (1.75 ± 0.59)	<0.001
HbA1c, mg/dL	6.67 ± 1.30	6.64 ± 1.37	6.48 ± 1.28	6.60 ± 1.31	6.58 ± 1.25	6.58 ± 1.30	6.53 ± 1.29	6.48 ± 1.14	6.55 ± 1.27	6.57 ± 1.05	0.301
LDL-C target goal											
Absolute goal	48 (51.6)	451 (42.1)	525 (43.9)	731 (47.8)	572 (52.3)	856 (58.0)	982 (59.4)	902 (60.7)	799 (63.4)	233 (57.4)	<0.001
Relative goal	31 (34.8)	202 (22.0)	240 (21.9)	457 (31.6)	370 (35.6)	472 (36.7)	572 (39.2)	550 (40.9)	470 (42.7)	128 (40.9)	<0.001
American goal	53 (59.6)	428 (46.6)	549 (50.0)	797 (55.1)	630 (60.6)	828 (64.4)	970 (66.4)	902 (67.1)	769 (69.8)	211 (67.4)	<0.001
European goal	23 (25.8)	161 (17.5)	185 (16.8)	356 (24.6)	289 (27.8)	402 (31.3)	473 (32.4)	474 (35.2)	404 (36.7)	109 (34.8)	<0.001

Values are presented as percentages (numbers) for categorical values and as means ± standard deviations for continuous values.

HbA1c, glycated hemoglobin; HDL-C, high-density lipoprotein cholesterol; KAMIR-NIH, Korea Acute Myocardial Infarction Registry-National Institutes of Health; KAMIR-V, Korea Acute Myocardial Infarction Registry-V; LDL-C, low-density lipoprotein cholesterol; LLA,

lipid-lowering agents; TC, total cholesterol; TG, triglyceride.
