# Supplementary material for: Ten-year trends in lipid management among patients after myocardial infarction in South Korea
Source: PLoS One. 2024 Oct 3;19(10):e0304710. doi: 10.1371/journal.pone.0304710 (PMC11449489; doi:10.1371/journal.pone.0304710)
Supplement: S3 Text — (PDF) [file pone.0304710.s003.pdf]

S3 Text. Associations between statin strategies and the incidence of LDL-C target goal attainment according to each Cox model among LLA-naïve participants.

	**Statin strategies**				**Model 1**	**Model 2**	**Model 3**	**Model 4**
	**No statin (N = 459)**	**Moderate-intensity statins (N = 5,929)**	**High-intensity statins (N = 6,373)**		**HR (95% CI)**	**HR (95% CI)**	**HR (95% CI)**	**HR (95% CI)**
Absolute LDL-C target goal	167 (36.4)	2,901 (48.9)	3,742 (58.7)	Moderate-intensity statins	1.85 (1.49-2.29)	1.86 (1.50-2.30)	1.49 (1.12-1.98)	2.13 (1.67-2.70)
				High-intensity statins	2.75 (2.22-3.40)	2.77 (2.24-3.43)	3.15 (2.37-4.19)	3.28 (2.58-4.17)
Relative LDL-C target goal	71 (19.2)	1,277 (24.1)	2,334 (40.4)	Moderate-intensity statins	1.49 (1.12-1.99)	1.49 (1.12-1.98)	1.41 (1.04-1.93)	1.32 (0.96-1.80)
				High-intensity statins	3.18 (2.40-4.23)	3.15 (2.37-4.19)	2.95 (2.17-4.01)	2.78 (2.03-3.80)
American goal	163 (44.2)	2,808 (53.1)	3,841 (66.4)	Moderate-intensity statins	1.58 (1.25-1.99)	1.59 (1.26-2.00)	1.70 (1.33-2.18)	1.71 (1.33-2.21)
				High-intensity statins	2.80 (2.23-3.53)	2.82 (2.24-3.55)	3.07 (2.40-3.94)	3.08 (2.39-3.98)
European goal	52 (14.1)	1,080 (20.4)	1,905 (32.9)	Moderate-intensity statins	1.72 (1.24-2.37)	1.71 (1.24-2.37)	1.65 (1.17-2.34)	1.60 (1.12-2.28)
				High-intensity statins	3.28 (2.38-4.51)	3.25 (2.36-4.48)	3.11 (2.20-4.40)	3.05 (2.15-4.34)

Model 1: Crude model.

Model 2: Adjusting for age and sex.

Model 3: Adjusting for all components in Model 2, plus use of EMS, Killip functional class, BMI, smoking status, past medical history, family history of CAD, and serum creatinine level.

Model 4: Adjusting for all components in Model 3 plus post-discharge medications, LVEF, PCI utilization, and a final diagnosis.

BMI, body mass index; CAD, coronary artery disease; CI, confidence interval; EMS, emergency medical service; HR, hazard ratio; LDL-C, low-density lipoprotein cholesterol; LLA, lipid-lowering agents; LVEF, left ventricular ejection fraction; PCI, percutaneous coronary intervention.
