# Supplementary material for: Ten-year trends in lipid management among patients after myocardial infarction in South Korea
Source: PLoS One. 2024 Oct 3;19(10):e0304710. doi: 10.1371/journal.pone.0304710 (PMC11449489; doi:10.1371/journal.pone.0304710)
Supplement: S4 Text — (PDF) [file pone.0304710.s004.pdf]

S4 Text. Proportions of prescriptions of post-discharge medications and PCI utilization according to Killip functional class, serum creatinine levels, and LVEF.

	**Killip functional class**			**Kidney function**			**LVEF**		
	**Killip I-II**	**Killip III-IV**	**p-value**	**sCr <1.5 mg/dL**	**sCr ≥1.5 mg/dL**	**p-value**	**LVEF ≥40%**	**LVEF <40%**	**p-value**
Aspirin	23,952 (99.5)	2,622 (99.6)	0.292	24,067 (99.5)	2,528 (99.3)	0.095	22,816 (99.5)	3,016 (99.6)	0.489
P2Y12 inhibitors	23,895 (99.2)	2,605 (99.0)	0.165	24,010 (99.3)	2,510 (98.5)	<.001	22,762 (99.3)	3,007 (99.3)	0.852
Beta-blockers	19,316 (80.2)	1,990 (75.6)	<.001	19,336 (79.9)	1,984 (77.9)	0.014	18,424 (80.4)	2,354 (77.7)	0.001
RAAS inhibitors	18,784 (78.0)	1,901 (72.2)	<.001	18,896 (78.1)	1,803 (70.8)	<.001	17,905 (78.1)	2,269 (74.9)	<.001
Statins	22,891 (95.1)	2,364 (89.8)	<.001	23,035 (95.2)	2,242 (88.0)	<.001	21,823 (95.2)	2,769 (91.4)	<.001
PCI utilization	22,142 (91.9)	2,362 (89.7)	<.001	22,318 (92.3)	2,207 (86.7)	<.001	21,173 (92.3)	2,695 (89.0)	<.001

Values are presented as percentages (numbers) for categorical values.

LVEF, left ventricular ejection fraction; PCI, percutaneous coronary intervention; RAAS, renin-angiotensin-aldosterone system; sCr, serum creatinine.
