# Supplementary material for: Ten-year trends in lipid management among patients after myocardial infarction in South Korea
Source: PLoS One. 2024 Oct 3;19(10):e0304710. doi: 10.1371/journal.pone.0304710 (PMC11449489; doi:10.1371/journal.pone.0304710)
Supplement: S5 Text — (PDF) [file pone.0304710.s005.pdf]

S5 Text. Temporal trends in lipid management in the study cohort (LDL <55 mg/dL, and LDL <55 mg/dL with relative goal).

	**The KAMIR-NIH registry (2011 – 2015)**					**The KAMIR-V registry (2016 – 2020)**					***P* for trend**
	**2011**	**2012**	**2013**	**2014**	**2015**	**2016**	**2017**	**2018**	**2019**	**2020**	
	**N=226**	**N=2,992**	**N=3,229**	**N=3,594**	**N=2,428**	**N=3,370**	**N=3,829**	**N=3,448**	**N=2,747**	**N=888**	
**LDL-C target goal**											
**LDL <55 mg/dL (1.4 mmol/L)**	23 (22.5)	204 (17.3)	251 (18.6)	378 (22.3)	282 (23.1)	481 (28.5)	558 (29.7)	519 (30.2)	478 (32.7)	146 (30.4)	<0.001
**LDL <55 mg/dL (1.4 mmol/L) with relative goal**	12 (12.2)	96 (9.5)	120 (9.7)	230 (14.4)	169 (14.6)	245 (16.7)	307 (18.4)	339 (21.8)	274 (21.4)	75 (20.4)	<0.001

Values are presented as percentages (numbers) for categorical values.

KAMIR-NIH, Korea Acute Myocardial Infarction Registry-National Institutes of Health; KAMIR-V, Korea Acute Myocardial Infarction Registry-V; LDL-C, low-density lipoprotein cholesterol.
