# Supplementary material for: Ten-year trends in lipid management among patients after myocardial infarction in South Korea
Source: PLoS One. 2024 Oct 3;19(10):e0304710. doi: 10.1371/journal.pone.0304710 (PMC11449489; doi:10.1371/journal.pone.0304710)

Trends In Lipid Management Among Korean Patients Following Myocardial Infarction

Temporal Trends in Prevalence of Dyslipidemia

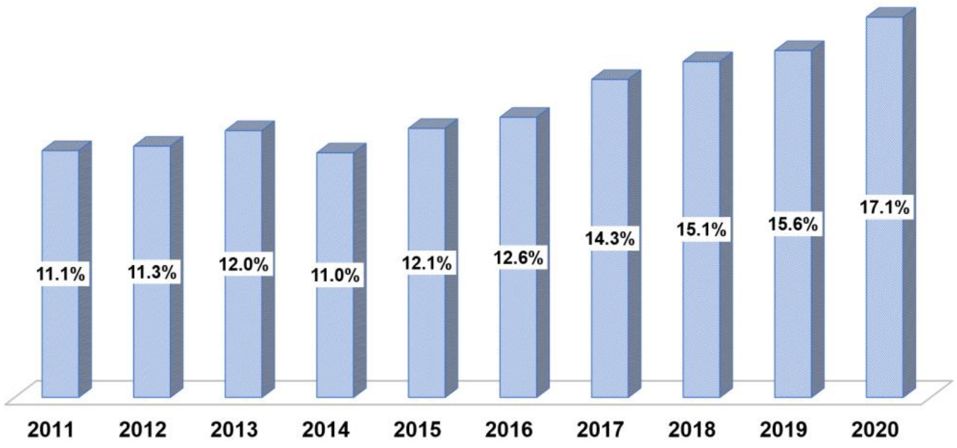

Prescription Fill Rates of High-Intensity Statins

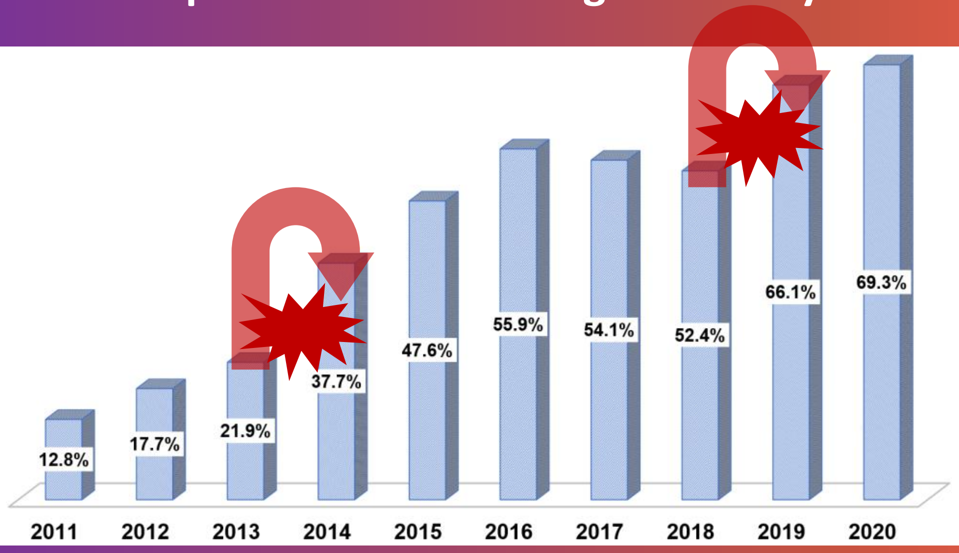

Temporal Trends in Achievement Rates of LDL-C Target Goals (LDL-C <70 mg/dL & >50% Reduction From Initial Levels)

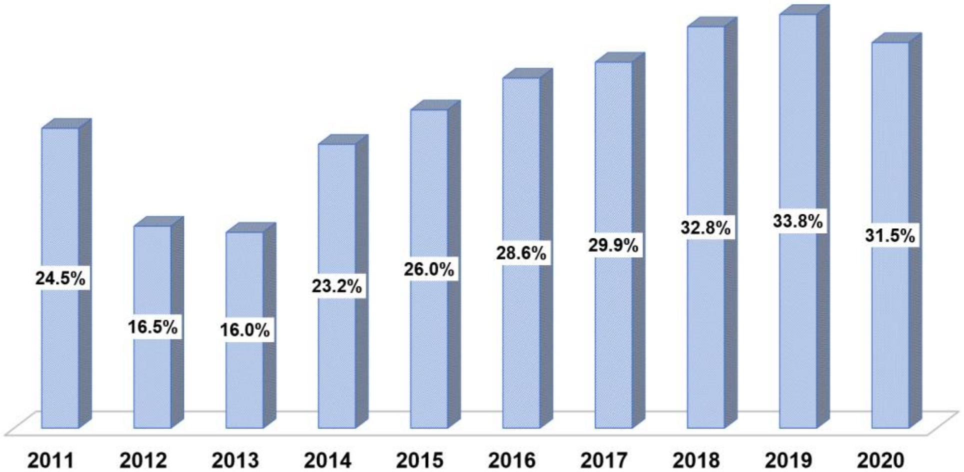

Adjusted Hazard Ratio in the Completely Adjusted Model

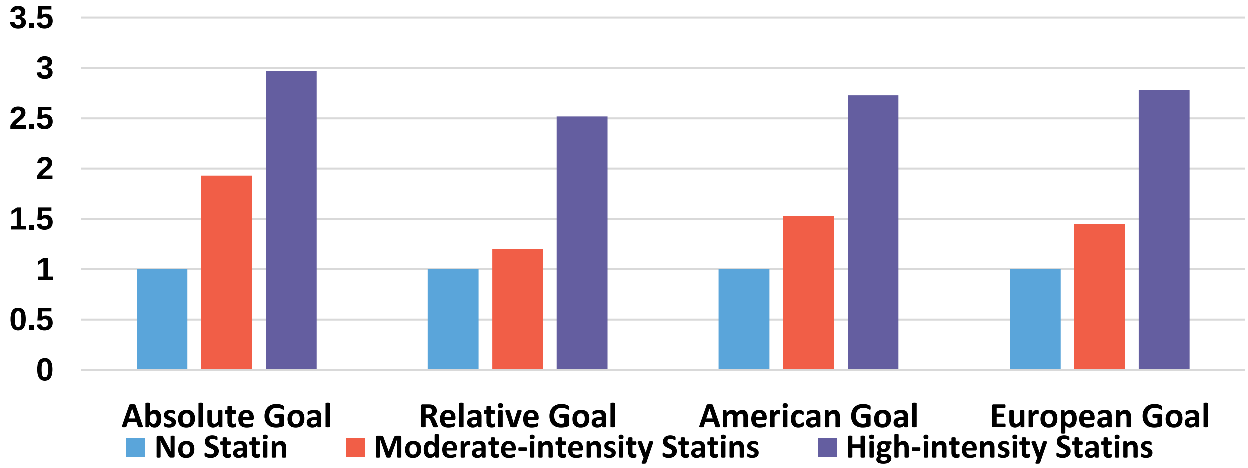

Supplement: S1 Graphical abstract — (PDF) [file pone.0304710.s006.pdf]
